# Buying time: a proof-of-concept randomized controlled trial to improve sleep quality and cognitive function among older adults with mild cognitive impairment

**DOI:** 10.1186/s13063-018-2837-7

**Published:** 2018-08-17

**Authors:** Ryan S. Falck, Jennifer C. Davis, John R. Best, Linda C. Li, Patrick C. Y. Chan, Anne B. Wyrough, Glenn J. Landry, Teresa Liu-Ambrose

**Affiliations:** 1 Department of Physical Therapy, Aging, Mobility and Cognitive Neuroscience Laboratory, Faculty of Medicine, University of British Columbia, Vancouver, BC Canada; 2 Department of Management, University of British Columbia – Okanagan Campus, Kelowna, BC Canada; 3 Department of Physical Therapy, Arthritis Research Canada, Faculty of Medicine, University of British Columbia, Vancouver, BC Canada

**Keywords:** Sleep, Cognitive function, Bright light therapy, Physical activity, Older adults

## Abstract

**Background:**

Current evidence suggests that good quality sleep is associated with preserved cognitive function and reduced dementia risk in older adults. Sleep complaints are especially common among older adults with mild cognitive impairment (MCI), and this may contribute to their increased risk for progression to dementia. Thus, improving their sleep may be important for maintaining their cognitive health. Chronotherapy is a set of intervention strategies that can improve sleep quality through strengthening the entrainment of the biological clock to the solar light-dark cycle, and includes strategies such as (1) bright light therapy (BLT); (2) physical activity (PA); and (3) good sleep hygiene. Of these strategies, BLT is the most potent and is based on providing individualized timing to entrain circadian rhythms. Thus, a personalized chronotherapy intervention of individually timed BLT and individually tailored PA promotion, in conjunction with general sleep hygiene education may promote older adult sleep quality. We therefore aim to carry out a proof-of-concept randomized controlled trial (RCT) to examine the efficacy of such a personalized chronotherapy intervention to improve sleep quality among older adults with MCI.

**Methods/design:**

This was a 24-week RCT of a personalized chronotherapy intervention aimed to primarily improve sleep quality as measured by the MotionWatch8©. Participants in the personalized chronotherapy group (INT) will receive four once-weekly, general sleep hygiene education classes, followed by 20 weeks of (1) individually timed BLT and (2) bi-weekly, individually tailored PA counseling phone calls in conjunction with receiving a consumer-available PA tracker—the Fitbit® Flex™. Ninety-six adults (aged 65–85 years) classified as having MCI (i.e., Mini-Mental State Exam (MMSE) ≥ 24; Montreal Cognitive Assessment (MoCA) ≤ 26; without dementia or significant functional impairment) will be randomized to either INT or a waitlist control group (CON).

**Discussion:**

The results of this trial will help determine if a personalized chronotherapy intervention that includes individually timed BLT and individually tailored PA promotion, along with general sleep hygiene education can promote sleep quality among older adults at increased risk for dementia. Our results will help inform best practices for promoting sleep quality among older adults with MCI.

**Trial registration:**

ClinicalTrials.gov, NCT02926157. Registered on 6 October 2016.

## Background

Worldwide, there is one new case of dementia detected every 4 s [1] and the costs of treating this epidemic are staggering. As of 2015 the estimated worldwide costs for dementia treatment were US$818 billion, and in 2018 the costs are expected to balloon to over US$1 trillion [2]. There is not yet a cure for dementia, and thus intervening with lifestyle strategies on known risk factors for cognitive impairment is an important strategy for reducing dementia risk—or at least delaying its onset [3].

Older adults with mild cognitive impairment (MCI) are at increased risk for dementia [4]. MCI is a clinical entity characterized by cognitive decline greater than expected for an individual’s age and education level, but which does not interfere notably with everyday function [5]. Importantly, 30% of older adults diagnosed with MCI develop dementia within 5 years [6], while in the same 5-year timespan only 2% of older adults without MCI are diagnosed with dementia [7]. There is currently a lack of effective pharmaceutical options for treating MCI, and thus lifestyle modifications to reshape the cognitive trajectory of older adults with MCI are an important line of scientific inquiry [8].

Improving older adult sleep quality is a promising strategy for maintaining older adult cognitive health. More than half of adults over 65 years report at least one chronic sleep complaint—the most common being the inability to stay asleep at night [9]. Poor sleep quality is also an important risk factor for Alzheimer’s disease (the most common form of dementia) [10], and older adults with MCI are more likely to experience poor sleep quality than healthy older adults [11]. Moreover, epidemiological evidence suggests poor sleep quality is associated with an increased risk of conversion from MCI to dementia [12], and thus improving sleep quality among individuals with MCI may help reduce dementia risk.

Sleep quality is closely tied to the function of circadian rhythms (i.e., ~ 24-h biological clock [13]), which coordinates physiology and behavior with the solar light-dark cycle [14–16]. Briefly, the process by which the biological clock is synchronized with the solar light-dark cycle is known as *entrainment* [17], and is regulated by the activity of the suprachiasmatic nuclei (SCN) which serves as “the master biological clock” of the central nervous system [18]. This process of entrainment occurs through certain external stimuli, known as *zeitgebers* (from the German *time-givers*), and helps to prevent inadvertent drifting or divergence from the 24-h day [19]. Of particular importance, aging is associated with (1) the biological clock initiating sleep-promoting mechanisms earlier in the day [20, 21]; and (2) decreased amplitude in circadian signals that increase sleep need [22, 23]. The weakening of circadian regulation that occurs with aging likely plays a prominent role in the fragmentation of sleep-wake rhythms observed in older adults during (1) the wake maintenance zone, which occurs 2–3 h before habitual bedtime and (2) the sleep maintenance zone, which occurs 2–3 h before habitual wake time [24]. Because aging appears to be linked to the divergence of the biological clock, chronotherapies that use effectively timed zeitgebers to help strengthen the entrainment of the SCN to the solar light-dark cycle may improve older adult sleep quality [25].

The principal entraining zeitgeber for the human biological clock is light [25, 26], which exerts its influence on blue-light-sensitive receptors in the retina [27]. Retinal light exposure directly stimulates greater activity of the SCN, which *phase delays* the biological clock such that the desire for sleep decreases and wakefulness increases (or is maintained); reduced retinal light exposure results in less activity of the SCN and increases the desire to sleep by *phase advancing* the biological clock [17]. While the importance of light is thus integral for the proper function of the SCN and the biological clock, older adults have reduced sensitivity to light, which leads to poorer function of the SCN and divergence of the biological clock from the solar light-dark cycle [28]. Behavioral changes in older adulthood—such as spending less time outdoors—could also further decrease bright light exposure, which may be a key factor in decreased amplitude of circadian rhythms [24]. Thus, older adults in particular may benefit from effectively timed bright light to strengthen the entrainment of the SCN to the solar light-dark cycle.

Bright light therapy (BLT) is an increasingly popular chronotherapy strategy [24]. While the efficacy of BLT as an intervention strategy is currently inconclusive [29, 30], the biological clock is not equally amenable to shifts at each phase in the circadian rhythm [17]. Indeed, any zeitgeber can cause the biological clock to phase advance, phase delay, or be entirely phase neutral depending on the biological clock time at which a zeitgeber is administered. As such, successful BLT requires an individualized approach where proper timing is essential [24].

Another potential zeitgeber for use as chronotherapy is physical activity (PA) [28, 31, 32]. Briefly, PA performed in the morning or early afternoon does not appear to have a consistent effect on phase shifts of the biological clock; however, engaging in PA in the late afternoon causes a phase advance of the biological clock, while late night PA causes phase delay of the biological clock [31, 32]. The time-based response to how PA can impact the SCN is hypothesized to coincide with the timing of the opening of the “sleep gate”—the shift of the biological clock from generating a waking signal that reduces sleep need, to generating a signal that facilitates sleep [33].

However, the use of PA in chronotherapy is challenging. The current evidence describing the effects of PA as a zeitgeber comes from controlled laboratory experiments, where the timing and intensity of PA in the form of exercise is tightly controlled. Conducting an intervention where participants would be asked to engage in regularly timed PA at a prescribed intensity would (1) be burdensome to participants and (2) require enormous resources to ensure participant adherence. More importantly, evidence suggests that regular PA—regardless of timing—is associated with better sleep quality [34, 35]. Less than 5% of older adults meet the current guidelines of 150 min/week of PA [36], and older adults with MCI are less active than their cognitively healthy peers [37]. Given that individually-timed BLT appears to be the most powerful chronotherapeutic [24], promoting older adult PA in conjunction with BLT may be a feasible approach to providing personalized chronotherapy.

In addition to the potential benefit of combining individually timed BLT with PA, improving sleep hygiene can positively impact sleep quality and aid chronotherapy [38]. Poor sleep hygiene exacerbates or even causes poor sleep, whereas good sleep hygiene results in feeling more rested and alert upon awakening—as well as a greater ability to function throughout the day. Sleep hygiene education, which teaches behavioral strategies to promote healthy sleep (e.g., avoiding watching television before bed), can also be useful as a strategy to promote behaviors which may improve circadian regulation—including light exposure and PA [39]. Current recommendations for improving older adult sleep quality therefore suggest combining (1) BLT; (2) PA; and (3) sleep hygiene education [40]. Importantly, preliminary evidence suggests combined BLT and PA in the form of exercise training, and sleep hygiene can improve sleep quality in older adults with insomnia [41].

While these results are promising, there remains a gap in our understanding of whether personalized chronotherapy is an effective approach to improving sleep quality among older adults with MCI. Thus, we propose a proof-of-concept randomized controlled trial (RCT) to examine the efficacy of a personalized chronotherapy intervention combining (1) individually timed BLT; (2) individually tailored PA promotion; and (3) general sleep hygiene education to improve the sleep quality of older adults with MCI. We will use a multimodal personalized chronotherapy intervention for improving the sleep quality of older adults with MCI, which may ultimately help in maintaining cognitive function and reduce dementia risk in this population.

## Methods

### Design

**Fig. 1:**
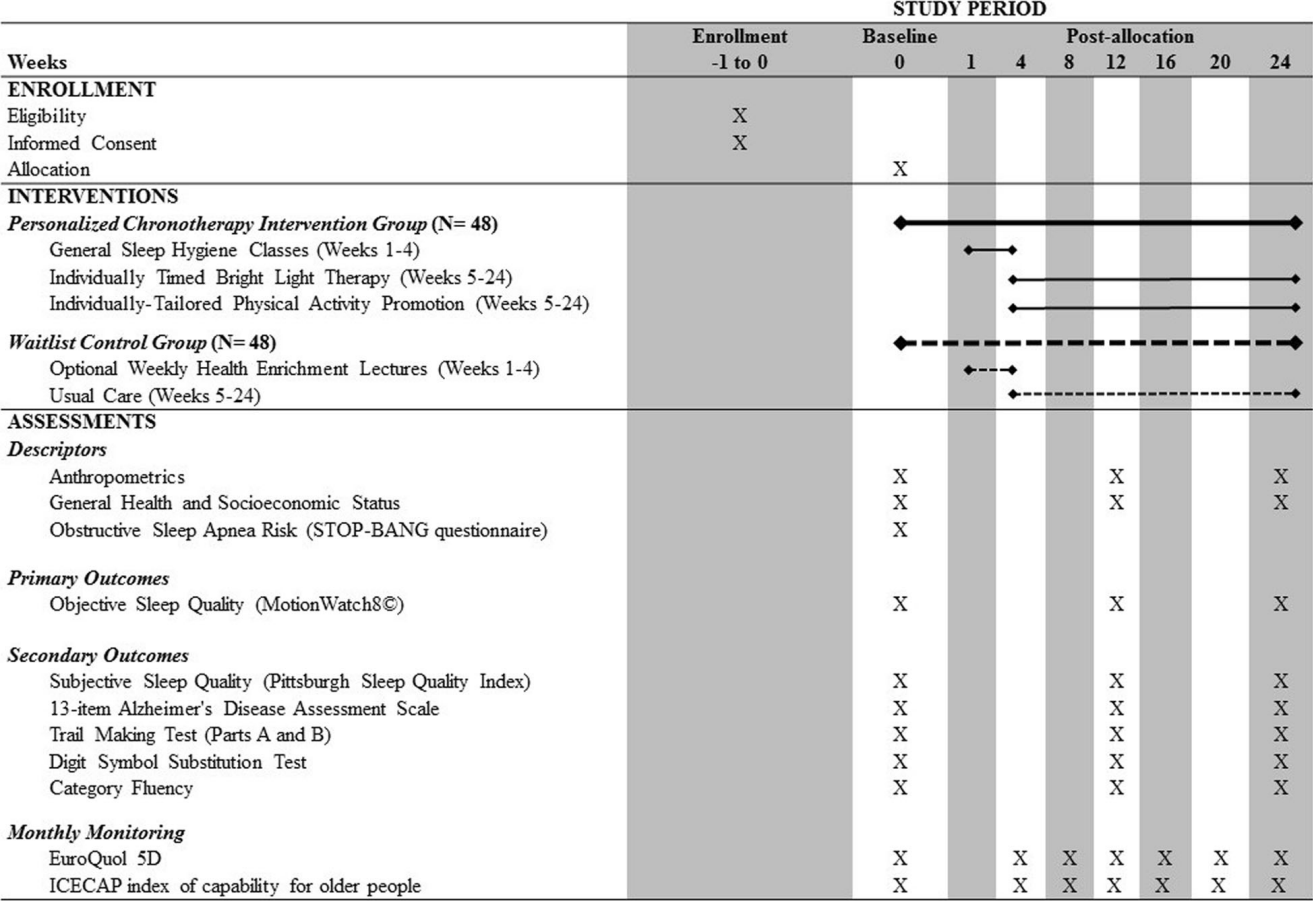
Standard protocol items: recommendation for interventional trials (SPIRIT) figure

We propose a proof-of-concept RCT of 96 community-dwelling older adults with MCI, aged 65 to 85 years. Participants randomized to the personalized chronotherapy group (INT) will receive the intervention over a 24-week period. There will be three measurement sessions with monthly monitoring (Fig. 1). Briefly, at each monthly monitoring participants will complete by telephone (1) the EuroQol 5D (EQ5D), a standardized instrument for measuring generic health status [42] and (2) the ICECAP index of capability for older people, which examines older adult quality of life [43]. Ethical approval has been obtained from the University of British Columbia's Clinical Research Ethics Board (H16-01029, 22 September 2016). All participants will provide a personally signed and dated informed consent document indicating that the individual has been informed of all pertinent aspects of the trial.

### Recruitment

We will recruit from the community and from our own database of individuals who have consented to be contacted about future research. Briefly, participants who have been previously involved in our research and have consented to being contacted about future research will be contacted. In addition, we will recruit participants by advertisements placed in local community centers, newspapers, and word-of-mouth referrals. Interested individuals will first be screened by telephone to check for general eligibility according to our criteria, and then screened using the Physical Activity Readiness Questionnaire (PAR-Q) [44]. Briefly, the PAR-Q is a simple screening tool for determining readiness to begin a PA program and for uncovering any potential health risks that may be associated with an increase in PA. Those who appear eligible will attend an information session.

### Eligibility

#### Inclusion criteria

We will include community-dwelling men and women who (1) are aged between 65 and 85 years and are living in their own home; (2) have preserved general cognition as indicated by a Mini-Mental State Examination (MMSE [45]) score ≥ 24/30; (3) have a baseline Montreal Cognitive Assessment (MoCA [46]) score < 26/30; (4) do not have dementia of any type; (5) do not have any significant impairment in daily function; (6) are currently experiencing poor sleep quality as indicated by a Pittsburgh Sleep Quality Index (PSQI [47]) score > 5; (7) scored < 5/15 on the 15-item Geriatric Depression Scale [48, 49]; (8) are able to walk independently and are in sufficient health to participate in regular PA as indicated by the PAR-Q [44]; and (9) understand, speak, and read English with acceptable visual and auditory acuity.

#### Exclusion criteria

We will exclude individuals who are (1) diagnosed with obstructive sleep apnea; (2) receiving continuous positive air pressure (CPAP) treatment; (3) at high risk for cardiac complications during PA or unable to self-regulate activity or to understand recommended PA level; and (4) have clinically important peripheral neuropathy or severe musculoskeletal or joint disease that impairs mobility.

### Sample size calculation

Our primary outcome measure is sleep efficiency as measured by the MotionWatch8© wrist-worn actigraphy unit (MW8) (CamNtech; Cambridge, UK) [50]. Sleep efficiency is one of several validated objective sleep parameters [51], and it is the gold standard in evaluating insomnia treatment efficiency [52]. Our pilot data suggested that sleep hygiene education alone had a small effect on sleep efficiency after 6 months (*d* = 0.16). As the proposed INT program is multifaceted (i.e., sleep hygiene education + individually timed BLT + individually tailored PA counseling), we anticipate a larger effect size in this RCT. Thus, assuming a two-sided alpha of 0.05, correlation across adjacent time points of 0.88 (estimated from pilot data), 40 participants per group will provide power of 0.85 to detect an effect size of 0.50 at the end of the intervention [53]. To allow for a drop-out rate of 15%, we will recruit a total of 96 older adults (i.e., 48 per group).

### Measurements

Baseline measurements will be obtained prior to randomization. There will be three measurement sessions: baseline, 12 weeks, and 24 weeks.

#### Descriptors

We will measure height using a wall-mounted stadiometer and measure weight using a calibrated digital scale. General health and socioeconomic status will be ascertained by questionnaire. In addition, we will survey participants at baseline for obstructive sleep apnea risk using the Snoring, Tiredness, Observed apnea, Blood pressure, Body mass index, Age, Neck circumference and Gender (STOP-BANG) questionnaire [54].

#### Primary outcome

We will measure sleep efficiency using the MW8. Briefly, the MW8 is a uni-axial, lightweight, wrist-worn accelerometer designed to observe acceleration ranging in magnitude from 0.01 to 8 G, with a frequency of 3–11 Hz. The filtered acceleration signal is digitized and the magnitude is summed over a user-specified time interval, or “epoch”. At the end of each interval, the summed value or activity “count” is stored in memory and the integrator is reset. Estimates of sleep quality parameters can then be extracted from the device based on the number of counts for a given epoch. For the present study, we will use 60-s epochs, which is consistent with current guidelines for estimating sleep quality [55].

Participants will first be fitted with the MW8 on the non-dominant wrist and provided detailed information on its features (i.e., the light sensor, event marker button, and status indicator). Participants will be instructed to press the event marker button each night when they start trying to sleep, and again each morning when they are finished trying to sleep. We will also provide participants with the Consensus Sleep Diary (CSD)—which they will be asked to complete upon awakening each morning [56]. After wearing the MW8 continuously for at least 14 days, participants will return the MW8 and completed CSD. We will subsequently download and analyze the MW8 data using MotionWare software 1.0.27 (CamNtech). The responses from the CSD will be used to confirm sleep windows identified by participants (as determined by time-stamped event markers). In cases where the event marker and CSD entries disagree for the start time of the sleep window, we will use the light sensor data to determine “lights out”. Similarly, when the event marker and CSD entry disagree for the end of the sleep window, we will use “lights on” and activity onset to determine the end of the sleep window. The MotionWare software will be used to estimate sleep efficiency (i.e., time asleep expressed as a percentage of time in bed).

#### Secondary outcomes

##### Objective sleep quality

In addition to estimating our primary outcome of sleep efficiency, we will use the MW8 and MotionWare software to estimate different parameters of sleep quality including: sleep duration (total time spent sleeping), sleep latency (time between “lights out” and falling asleep), and fragmentation index. Briefly, fragmentation index is defined by MotionWare as the sum of (1) the total time spent sleeping categorized as mobile in the epoch-by-epoch mobile/immobile categorization expressed as a percentage of the time spent asleep and (2) the number of immobile bouts that were ≤ 1 min in length expressed as a percentage of the total number of immobile bouts during time spent sleeping. The MW8 provides validated estimates for measures of sleep quality for the following indices: sleep duration, efficiency, fragmentation, and latency [50, 57].

##### Subjective sleep quality

We will also use the PSQI as a measure of subjective sleep quality [47]. This 19-item questionnaire assesses sleep quality using subjective ratings based on the summation of 7 different component scores (i.e., sleep quality, sleep latency, sleep duration, habitual sleep efficiency, sleep disturbance, use of sleeping medication, and daytime dysfunction). Respondents will be asked to answer the questionnaire retrospectively, surveying sleep components spanning the previous month.

##### Cognitive function

We will use a comprehensive neuropsychological battery to examine changes in cognitive function including (1) the 13-item Alzheimer’s Disease Assessment Scale [58], (2) Trail Making (parts A and B) [59]; (3) the Digit Symbol Substitution Test [60]; and (4) category fluency (i.e., vegetables) [60].

### Treatment allocation

#### Randomization

Participants will be randomly assigned (1:1) to either the INT or the waitlist control group (CON). We will use permuted blocks of varying size to ensure balance over time. To ensure concealment of the treatment allocation, the randomization sequences will be generated and held by a central web-based randomization service.

#### Allocation concealment

Recruitment and enrollment of participants will be managed by the research coordinator who will screen for study eligibility, obtain informed consent, and conduct baseline assessment. Following completion of baseline assessment, the research coordinator will access the web-based randomization service and the participant will be assigned a participant number and allocated to the INT or the CON group. Research personnel performing the outcome assessment and data analysis will be blinded to group allocation. We will not be able to blind participants or personnel delivering the interventions to group allocation.

### Experimental groups

#### Personalized chronotherapy group (INT)

Participants allocated to the INT group will receive four once-weekly general sleep hygiene education classes, followed by 20 weeks of (1) individually timed BLT based on the participant’s sleep profile and (2) individually tailored PA promotion.

##### General sleep hygiene education

Following baseline assessment, participants in the INT group will receive a 4-week general sleep hygiene education course (weeks 1–4; 1×/week; 2 h/session). These courses will provide participants with an understanding of (1) how sleep is regulated; (2) the relationship between sleep and cognition; (3) how sleep changes as we age; and (4) why preserving sleep quality is fundamental to healthy aging. Participants will learn strategies to protect sleep including the importance of individually timed BLT (i.e., seek bright light exposure during the day, while avoiding light at night) and regular PA.

At the completion of the 4-week sleep hygiene education course, INT group participants will receive 20 weeks of (1) individually timed BLT based on the participant’s sleep profile and (2) individually tailored PA promotion.

##### Individually timed bright light therapy (BLT)

In week 5, participants in the INT group will meet with a trained research assistant to develop a daily BLT schedule designed to improve sleep quality and address sleep complaints. Using the results from the STOP-BANG questionnaire, we will first counsel participants with moderate-to-high risk of sleep apnea to see a physician. The participant will then work with the research assistant to determine the best regularly scheduled bed time and wake time in order for the participant to dedicate 8 h to sleeping each night (i.e., sleep window). Using the results from the MW8 sleep recordings (i.e., the participant’s current sleep window), and the participant’s recommended sleep window, we will classify participants as (1) phase advanced (falls asleep earlier than intended); (2) phase delayed (falls asleep later than intended or wakes up later than intended); or (3) phase neutral (falls asleep when intended). Participants will then be provided with a commercially available BLT device (Philips goLITE BLU) and be provided a personalized BLT schedule based on their phase classification. The goLITE BLU emits short-wave blue light (~ 200 lx), which possesses greater phase-shifting properties than the rest of the visible light spectrum [61–64]. All BLT doses will be 1 h in duration, twice daily for 20 weeks (i.e., week 5–24). The time and dose-dependent effects of BLT have been well-described elsewhere [65–69].

**Phase advanced:** Participants will be counseled to use BLT 2 h after waking, and again 11 h later (e.g., morning BLT dose, 8:00–9:00 a.m.; evening BLT dose, 7:00–8:00 p.m.). Participants will be instructed to avoid light 1.5 h before sleep window onset. Participants can otherwise continue their normal light routine at night.

**Phase delayed:** Participants will be counseled to use BLT 30 min after waking, and again 11 h later. Participants will be counseled to avoid all light 3 h before sleep window onset.

**Phase neutral:** participants with a neutral sleep phase will be further sub-categorized based on their current waking routine.

Participants who wake up earlier than intended will be counseled to use BLT 2 h after waking, and again 11 h later. Participants will be counseled to avoid light 1.5 h before sleep window onset. Participants can otherwise continue their normal light routine at night.

Participants who wake up when intended will be counseled to use BLT 1 h after waking, and again 11 h later. Participants will be instructed to avoid light beginning 2 h before sleep window onset. Participants can otherwise continue their normal light routine at night.

##### Individually tailored physical activity (PA) promotion

INT group participants will also meet with a certified fitness professional (i.e., the coach) in week 5 to review their current PA level, and develop an individualized PA plan. We will use the brief action planning approach to help participants develop their individualized PA plan [70]. Briefly, this approach requires the coach to guide participants to (1) set an activity goal; (2) develop an action plan; (3) identify barriers and solutions; and then (4) rate their confidence in the plan. This process will be repeated until the confidence rating reaches > 7/10, indicating that the person is confident about implementing the plan. Participants will be counseled to engage in PA at least 4 h before their regularly scheduled bed time.

Participants will then be provided a Fitbit® Flex™ to be worn on the non-dominant wrist 24 h/day—except during water-based activity or when charging the device. The Fitbit® Flex™ is one of the most common wireless PA trackers in the consumer market and is capable of collecting PA data, uploading it to the web, and producing simple graphs and charts of an individual’s activity. PA data from the device will be wirelessly synchronized with the Fitbit online Dashboard, which can be viewed only by the participants and their fitness professional. During weeks 5–24, the fitness professional will review the individual’s PA on the Dashboard and progressively modify the activity goals during nine bi-weekly phone calls.

#### Waitlist group (CON)

Participants in the CON group will receive access to four optional weekly health enrichment lectures during weeks 1–4 of the intervention period. These lectures will provide educational material for participants on healthy weight management, diet, mindful meditation, and goal setting. This group will not receive specific information on PA during these lectures. During weeks 5–24, participants will not receive any intervention and will go about their usual activity. After 6 months follow up, CON participants will receive (1) the sleep hygiene education course and (2) PA counseling and a Fitbit® Flex™.

### Intervention compliance and compliance measures

For INT participants, we will track compliance to both the BLT prescription and PA promotion program. Briefly, INT participants will receive monthly calendars monitoring their BLT use, which they will complete and send by mail. Monthly BLT calendars will be reviewed upon receipt from participants and we will follow up with INT participants who do not appear to be following their BLT prescription (i.e., < 50% compliance or did not mail their most recent monthly BLT calendar). We will also monitor weekly PA for each INT participant using the Fitbit online Dashboard, and we will follow up with participants who do not appear to be logging their PA using their Fitbt (i.e., no PA minutes logged on the Fitbit Dashboard within the past 2 weeks). As an additional measure to increase participant compliance and understanding, we will maintain a bi-weekly phone call schedule with participants and will follow up on all missed appointments or missed phone calls.

### Adverse events monitoring

We will have a Data Safety Monitoring Committee (Teresa Liu-Ambrose, Jennifer C. Davis, and Linda Li). All adverse events will be reviewed by the monitoring board. They will stop the study if the adverse event data demonstrate any hazards directly due to the intervention (e.g., increased falls rate) based on monthly report.

### Statistical analyses

The primary objective of this study is to provide evidence for the efficacy of a personalized chronotherapy intervention in improving sleep quality. Our primary analyses will therefore follow the intention-to-treat principle (i.e., all individuals will be analyzed according to their group allocation regardless of compliance). We will evaluate between-group differences (INT vs. CON) in sleep quality using mixed linear models. Maximum likelihood estimation will be used in order to include all randomized participants to estimate treatment effects, regardless of loss to follow up. Time will be considered as a repeated, categorical variable and will be included as a fixed effect in addition to group and group-by-time interaction. The intercept will be specified as a random effect. Primary and secondary outcomes will be analyzed using this same analytic model.

## Discussion

Our research team will use a multi-pronged approach to explore the utility of personalized chronotherapy to improve sleep quality and cognitive function among older adults with MCI. This proposed trial may have important public health and mechanistic implications.

### Public health

Current guidelines for sleep recommend older adults should sleep 7–8 h/night [71]. Given that older adults with MCI experience poor sleep [24], our study may provide an evidence-based chronotherapy approach to improving sleep quality in older adults with MCI. Our approach to improving sleep quality in older adults with MCI may also help maintain the cognitive health of these individuals at risk for dementia.

### Mechanistic

Several lifestyle approaches to maintaining the cognitive health of older adults with MCI have been proposed such as (1) exercise [72] and (2) cognitive training [73]. However, no study has yet examined if changes in sleep quality can improve cognitive health among older adults with MCI. Should our intervention prove to be efficacious at improving cognitive function through improved sleep quality, it would be a major contribution towards our understanding of how to maintain the cognitive health of older adults with MCI.

### Trial status

At 1 October 2017 we have obtained ethical approval, have registered the trial, and have successfully recruited 38 participants.
